# Epigenetic Regulation of NKT-Cell-Related Gene Signatures and Prognostic Implications in Oropharyngeal Squamous Cell Carcinoma

**DOI:** 10.3390/cancers17223666

**Published:** 2025-11-15

**Authors:** Luka Minarik, Rita Khoueiry, Mirela Leskur, Vincent Cahais, Zdenko Herceg, Merica Glavina Durdov, Benjamin Benzon

**Affiliations:** 1 Department of ENT, County Hospital Čakovec, 40000 Čakovec, Croatia; 2 Epigenomics and Mechanisms Branch, International Agency for Research on Cancer, 69366 Lyon, France; 3 Department of Biochemistry and Medical Chemistry, University of Split School of Medicine, 21000 Split, Croatia; 4 Department of Pathology, Forensic Medicine and Cytology, University Hospital of Split, 21000 Split, Croatia; 5 Department of Pathology, University of Split School of Medicine, 21000 Split, Croatia; 6 Department of Anatomy, Histology and Embryology, University of Split School of Medicine, 21000 Split, Croatia; benjamin.benzon@mefst.hr; 7 Department of Anatomy, University of Mostar School of Medicine, 88000 Mostar, Bosnia and Herzegovina

**Keywords:** immune microenvironment, ITK, ATF2, ZNF683, methylation

## Abstract

Background: Oropharyngeal squamous cell carcinoma (OPSCC) is a major subtype of head and neck cancer, with prognosis increasingly influenced by the tumour immune microenvironment. Although immune checkpoint inhibitors have improved outcomes for some patients, reliable predictive biomarkers remain limited. Methods: This study aimed to investigate the prognostic relevance and epigenetic regulation of natural killer T (NKT)-cell-related gene signatures in OPSCC. Clinicopathological and transcriptomic data from 81 OPSCC patients were analysed using single-sample gene set enrichment analysis (ssGSEA) to evaluate immune-related gene set enrichment scores. Associations with overall survival and clinical features were assessed, and candidate prognostic genes were further explored through expression, methylation, and network analyses. Results: High NKT cell differentiation enrichment scores were significantly associated with improved survival and favourable clinical features. Gene-level analyses identified ITK, ZNF683, and ATF2 as key prognostic markers linked to T-cell signalling and epigenetic regulation. Methylation profiling revealed hypermethylation of ITK and hypomethylation of ZNF683 in tumour tissues, suggesting an epigenetic basis for altered gene expression. Conclusions: These findings highlight NKT cell differentiation as a strong prognostic indicator in OPSCC and support further exploration of epigenetic–immunologic interactions as potential therapeutic targets.

## Simple Summary

This study investigates the role of natural killer T (NKT)-cell-related gene signatures and their epigenetic regulation in oropharyngeal squamous cell carcinoma (OPSCC). Transcriptomic data from 81 OPSCC patients were analysed using single-sample gene set enrichment analysis (ssGSEA), revealing that high NKT cell differentiation scores were significantly associated with improved overall survival. Three genes (ITK, ZNF683, and ATF2) emerged as key prognostic markers linked to immune activation and T-cell signalling pathways. Methylation analyses from TCGA and GEO datasets indicated hypermethylation of ITK and hypomethylation of ZNF683 in tumour samples, suggesting epigenetic regulation of these immune-related genes. The immune microenvironment showed predominant T regulatory cells, macrophages, and neutrophils, alongside high expression of IL-15, IL-18, TNFα, and TGFβ. These findings highlight NKT-cell-associated gene regulation as a potential prognostic and therapeutic target in OPSCC, emphasising the interplay between epigenetics and antitumor immunity.

## 1. Introduction

Head and neck cancers (HNCs) represent the fifth most common cancer worldwide, making roughly 4% of all cancer cases, with a yearly incidence of around 946,000 estimated cases [1]. Oropharyngeal cancer makes up about 23% of all HNC, with oropharyngeal squamous cancer (OPSCC) being the most common histological type [2,3]. While tobacco and alcohol consumption remain significant risk factors in many developing regions, the rising incidence of human papillomavirus (HPV) associated OPSCC in high-income countries has markedly altered both clinical presentation and prognosis [4,5]. Reflecting their clinical, genetical, and immunological differences, the American Joint Committee on Cancer (AJCC) introduced distinct staging criteria for HPV-positive and HPV-negative OPSCC in its eighth edition, recognising their differences in biological and clinical behaviours [6].

Traditionally, HNC are treated surgically with radiotherapy and platinum-based chemotherapy. However, recently, the new paradigm for cancer therapy is based on immune checkpoint inhibition, especially targeted therapy of the PD-1/PD-L1 axis and CTLA-4 [7]. This headway in cancer therapy significantly changed the way we understand OPSCC. Therapies such as pembrolizumab and nivolumab, targeting the PD-1/PD-L1 axis, have demonstrated improved survival in recurrent and metastatic HNSCC, including OPSCC, in pivotal trials like CheckMate-141, KEYNOTE-012, and KEYNOTE-040 [8,9,10]. These advances mark a paradigm shift in oncologic care, moving toward more personalised and immune-targeted approaches.

Despite initial clinical success, immunotherapy faces many challenges—one of them being that a substantial proportion of patients do not respond to immunotherapy, which may partly be explained by differences in the tumour microenvironment (TME) and biomarker expression. In addition, PD-L1 expression, tumour mutational burden (TMB), and the presence of specific T cells represent potential predictive biomarkers, but none have yet been universally accepted [11,12]. Furthermore, mechanisms of resistance are present, either innate (e.g., loss of MHC expression) or acquired during therapy [13]. This has led to an intensified search for additional indicators of immune responsiveness and tumour progression.

Given the complex interplay between tumour cells and the surrounding immune landscape, there is increasing interest in leveraging transcriptomic data to better characterise the tumour immune microenvironment (TIM) and identify immune-related gene signatures associated with disease progression and outcome [14,15]. By capturing the expression of immune-related gene signatures at the bulk or single-sample level, research can be conducted to identify patterns associated with immune infiltration, suppression, or activation. Single-sample gene set enrichment analysis (ssGSEA) is a powerful computational method that allows for the quantification of enrichment scores for specific gene sets in individual samples, enabling a more granular analysis of immune cell activity and signalling pathways [14,16]. This approach has been used to dissect the roles of various immune subsets including neutrophils, T cells, B cells, macrophages, and natural killer (NK) cells in tumour biology [16].

## 2. Materials and Methods

Clinicopathological, methylation and transcriptomic data from patients diagnosed with OPSCC were retrieved from the publicly available cBioPortal for Cancer Genomics database (https://www.cbioportal.org/, “Head and Neck Squamous Cell Carcinoma (TCGA, Firehose Legacy)”, accessed on the 23 December 2024). Out of a list of 530 patients diagnosed with HNC, we filtered 81 patients whose cancers were anatomically located in the oropharynx. No prior sample size calculation was performed. Gene expression profiles were analysed using ssGSEA, implemented via the GenePattern online platform (https://www.genepattern.org/), to assess the enrichment of immune-related pathways. Specifically, gene sets corresponding to activation, differentiation, and proliferation pathways of B cells, T cells, macrophages, neutrophils, NK cells, and NKT cells were evaluated. All gene sets were downloaded from the Molecular Signatures Database (MSigDB, v2024.1, https://www.gsea-msigdb.org/). The full list of immune-related pathways is available in Table 1. Survival analysis was performed for each selected immune-related pathway using Kaplan–Meier (KM) survival curves and log-rank tests. To evaluate the independent prognostic effect of NKT cell differentiation enrichment, a discrete-time survival model using a complementary log-log (cloglog) link was fitted. Bias-reduced (Firth-like) estimates were calculated to minimise small-sample bias. Covariates included HPV status (positive vs. negative) and smoking history (yes vs. no). Hazard ratios (HRs) and 95% confidence intervals (CIs) were reported on the log scale. Pathways found to be significantly associated with patient outcomes were further correlated with clinicopathological variables, including tumour grade, clinical stage, p16 status, tobacco, and alcohol consumption status. Gene expression data were downloaded as log2-transformed RNA-Seq V2 RSEM values from TCGA. Expression matrices were normalised using Z-score transformation across samples to mitigate inter-sample variability. We converted gene Z-scores into fold change values by normalising them to the median expression level of the lowest expression category. To explore the downstream molecular mechanisms associated with NKT cell-related genes, KEGG pathway enrichment analysis was performed. Genes significantly correlated with NKT differentiation ES were used as input. Pathways with an adjusted *p* value < 0.05 were considered significantly enriched. A gene–pathway interaction network was subsequently generated to visualise functional associations between key genes and their corresponding KEGG pathways. Searching the GEO DataSet, we collected methylation profiling data from prior studies performed on oropharyngeal cancer that uploaded data using Illumina HumanMethylation450 BeadChip (Illumina, San Diego, CA, USA). For comparing continuous variables, ANOVA or *t* test was used, with Welch correction if necessary. Correlations between continuous variables were performed using Spearman’s method if necessary; for reduction in dimension, principal component analysis was used. Coefficients of correlation were interpreted as suggested by Chan [17]. Statistical significance was defined as a *p* value less than 0.05. All statistical analyses were performed using GraphPad Prism 10 (GraphPad software, La Jolla, CA, USA, and R (v. 2024.12.1+563)).

## 3. Results

### 3.1. Transcriptomic Analysis of Immune-Related Gene Sets

**Figure 1:**
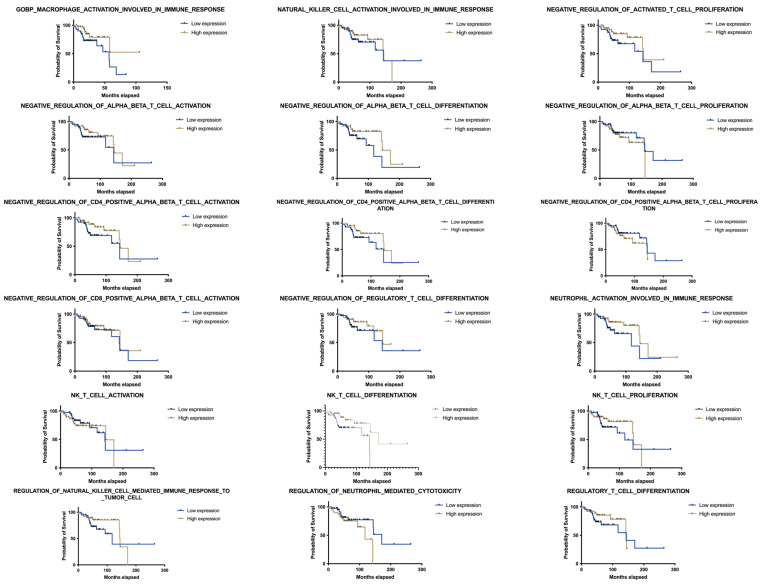
Kaplan–Meier survival analysis of NKT cell differentiation gene set enrichment and patient OS.

Transcriptomic analysis of 81 OPSCC patients revealed differential enrichment of various immune-related gene sets. Among the immune pathways analysed using ssGSEA, NKT cell differentiation exhibited the strongest association with overall survival (OS). Patients with high NKT enrichment scores (ESs) demonstrated significantly improved OS compared to those with lower scores (median survival: 5.62 vs. 4.68 years, *p* = 0.015; Figure 1, Table 1). KM analysis of additional immune-related gene sets showed variable survival trends, but none reached statistical significance after multiple testing correction, highlighting the unique prognostic relevance of the NKT cell signature.

In a multivariate discrete-time survival model adjusting for HPV status and smoking, high NKT differentiation remained an independent predictor of improved overall survival (HR = 0.13, 95% CI 0.03–0.49, *p* = 0.003, Table 2). Neither HPV positivity (HR = 1.34, 95% CI 0.43–4.17, *p* = 0.612) nor smoking status (HR = 0.69, 95% CI 0.29–1.66, *p* = 0.410) were significantly associated with survival. These results confirm that NKT cell differentiation enrichment contributes prognostic value independently of major clinical confounders.

### 3.2. Clinical Data and NKT Cell Differentiation

**Figure 2:**
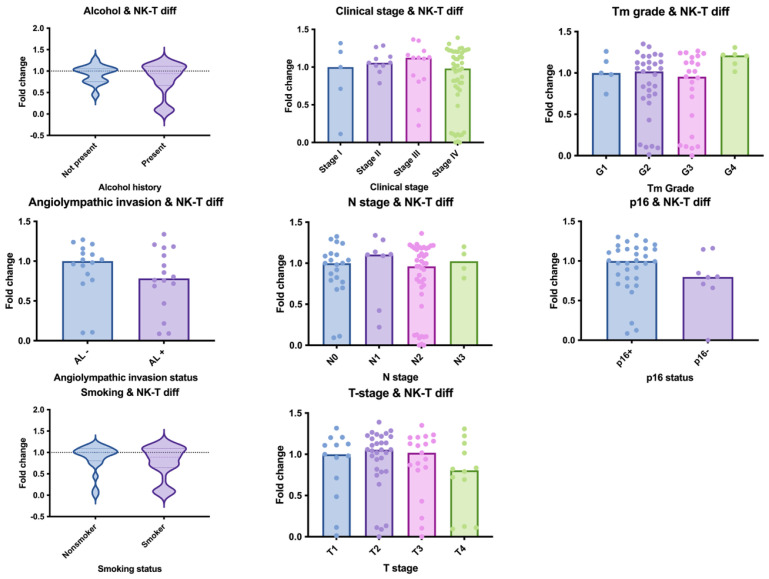
Association of NKT cell differentiation ES with clinicopathological features in OPSCC. Indicidual patients’ scores are represented as dots. Patients with no alcohol history demonstrated significantly higher NKT cell differentiation ES compared to those with alcohol use. Similarly, earlier clinical stage and grade was associated with increased NKT differentiation. Data are presented as mean ± standard deviation (SD). Dots represent individual patients.

High NKT cell differentiation ESs were significantly associated with favourable clinicopathological features. Specifically, patients in the high ES group were more likely to have lower tumour grade (*p* = 0.0079), earlier clinical stage (*p* = 0.011), and negative history of alcohol consumption (*p* = 0.0202) (Figure 2). No statistically significant associations were observed between NKT cell ES and tobacco consumption or p16 status.

### 3.3. Gene Expression and Methylation

**Figure 3:**
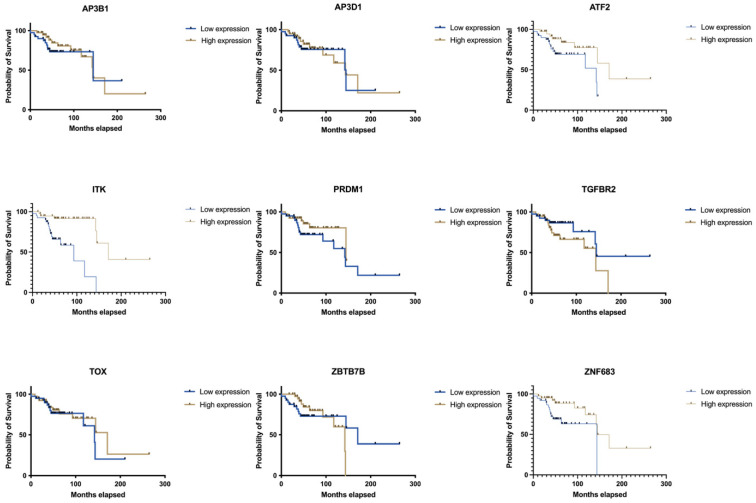
Kaplan–Meier survival analysis of candidate genes within the NKT cell differentiation signature in OPSCC. Overall survival was compared between patients with low versus high median expression of AP3B1, AP3D1, ATF2, ITK, PRDM1, TGFBR2, TOX, ZBTB7B, and ZNF683. Among these, ITK, ZNF683, and ATF2 expression were significantly associated with improved overall survival. Other genes in the signature did not demonstrate statistically significant associations. High-risk and low-risk groups were stratified by median expression. Survival differences were assessed using the log-rank test.

Further analysis of gene expression within the NKT differentiation gene set identified *ITK*, *ATF2*, and *ZNF683* as potential drivers of the observed prognostic signal. Among these, *ITK* expression was most strongly correlated with improved overall survival (*p* < 0.01), suggesting it may play a role in the antitumour immune activity of NKT cells in OPSCC (Figure 3, Table 3).

**Figure 4:**
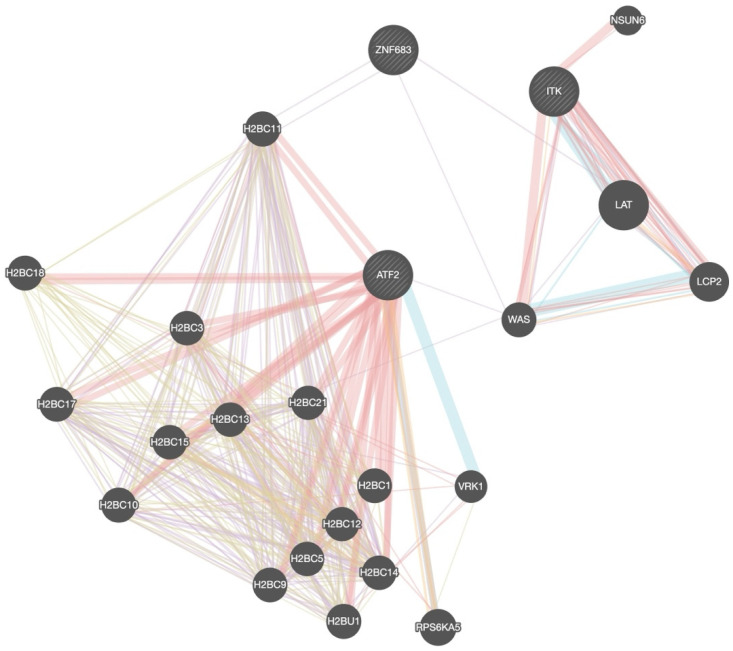
Gene–gene interaction network of NKT cell differentiation-associated prognostic genes in OPSCC. The red color indicates physical interactions, purple indicates co-expression, blue for pathway interactions and brown for shared protein domains.

To explore the potential interactions and functional context of these genes, we utilized the GeneMANIA network analysis tool. The interaction network (Figure 4) revealed that *ITK* is closely connected to canonical T-cell signalling mediators such as *LAT*, *WAS*, and *LCP2*, indicating its central role in immune signal transduction. Similarly, *ATF2* showed strong interaction with numerous histone H2B family members (e.g., H2BC11, *H2BC13*, *H2BC21*), confirming epigenetic or transcriptional regulatory roles. *ZNF683*, while less interconnected, clustered in proximity with *ITK* and *ATF2*, supporting its shared involvement in immune regulatory pathways.

These findings suggest that the prognostic signal associated with NKT cell differentiation may be driven, at least in part, by the coordinated activity of transcriptional regulators (*ATF2*) and TCR-associated proteins (*ZNF683*, *ITK*), implicating both epigenetic control and T-cell activation in the modulation of OPSCC progression.

**Figure 5:**
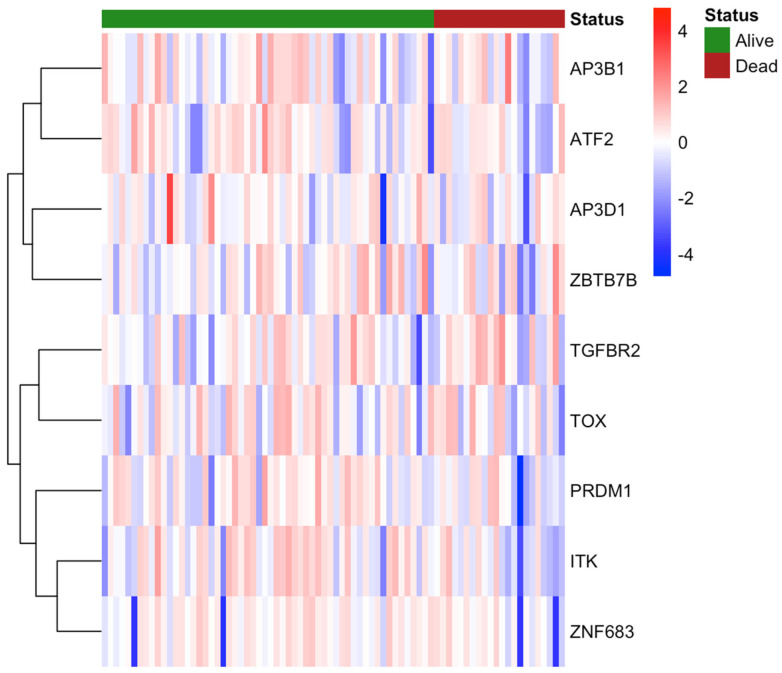
Heatmap of NKT cell differentiation gene set expression in OPSCC. The heatmap illustrates Z-scores of genes within the NKT cell differentiation signature across the OPSCC cohort. Rows represent individual genes, while columns correspond to patient samples.

**Figure 6:**
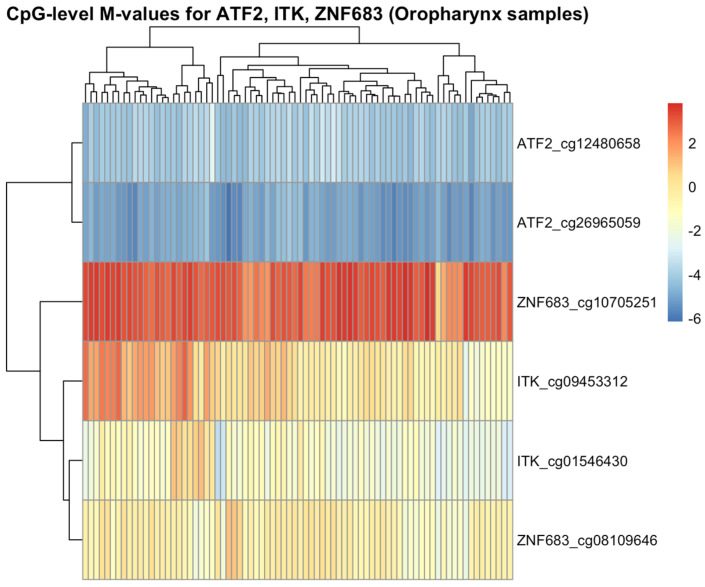
Heatmap presenting significant CpG methylation profiles of ATF2, ITK, and ZNF683 in OPSCC from TCGA. Here we can see an overall hypomethylation for the ATF2 gene, strong hypermethylation of ZNF683, and intermediate methylation for ITK.

**Figure 7:**
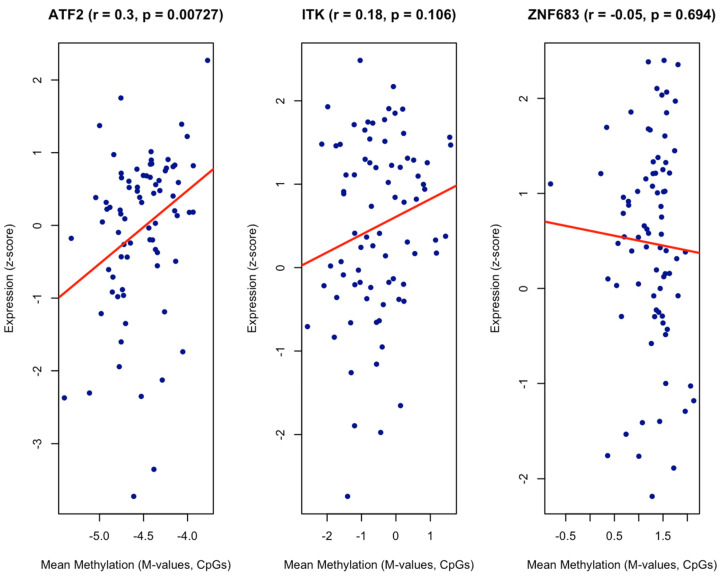
Correlation between mean CpG methylation and gene expression of ATF2, ITK, and ZNF683 in OPSCC.

Because of the potential epigenetic changes, we analysed the methylation data from our samples and correlated them with transcriptomic data of our three genes of interest (Figure 5). While CpG sites annotated to ATF2 were overall hypomethylated across oropharyngeal cancer samples (Figure 6), mean methylation levels showed a significant positive correlation (r = 0.03, *p* = 0.007) with gene expression (Figure 7).

**Figure 8:**
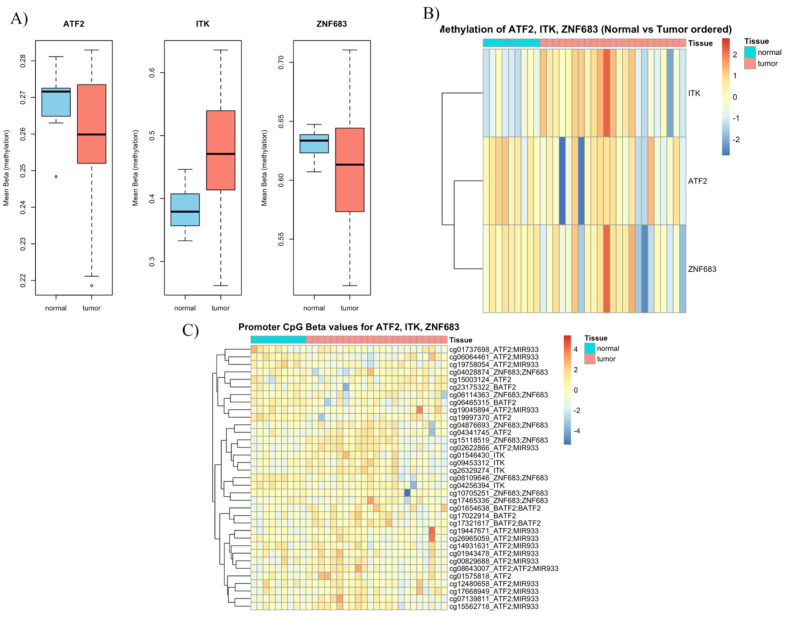
Analysis of data collected from Soares-Lima et al. (**A**) Differential DNA methylation of ATF2, ITK, and ZNF683 in OPSCC versus normal tissue. Boxplots depict mean beta values (DNA methylation levels) for ATF2, ITK, and ZNF683 in normal versus tumour samples. (**B**) Heatmap of DNA methylation patterns in ATF2, ITK, and ZNF683 across OPSCC and normal tissue. (**C**) Heatmap of CpG methylation β-values in ATF2, ITK, and ZNF683 loci in OPSCC tumour and mucosa samples [18].

To further investigate the role of epigenetics in these specific genes, we analysed methylation profile data from two studies that published their methylation meta-data (GEO access: GSE178219 and GSE98807) [18,19]. When analysing the data from Soares-Lima et al., we found that ITK was hypermethylated compared to normal tissue (*p* = 0.001) (Figure 8A). ZNF683 promotors were hypomethylated (*p* = 0.08) and ATF2 promotor methylation scores were similar in both groups (*p* = 0.07) (Figure 8B, Table 4). We extracted the CpG promotors and their methylation status for the three genes of interest (Figure 8C).

**Figure 9:**
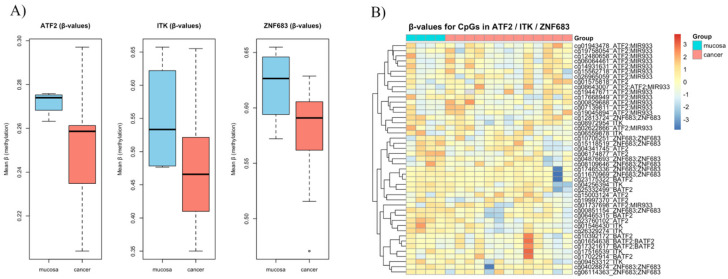
Data analysis from Nakagawa et al. (**A**) Comparison of DNA methylation β-values for ATF2, ITK, and ZNF683 in normal mucosa versus OPSCC tumour tissue. (**B**) Heatmap of CpG methylation β-values in ATF2, ITK, and ZNF683 loci in OPSCC tumour and mucosa samples [19].

**Figure 10:**
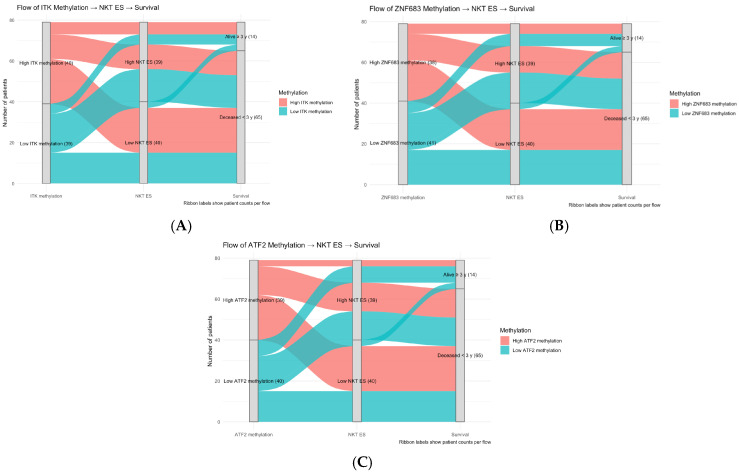
Sankey plots showing the distribution of patients based on low and high gene methylation distribution of selected genes, NKT differentiation ES scores, and survival status. ITK (**A**), ZNF683 (**B**) and ATF2 (**C**), all showed that higher methylation groups had a lower NKT ES, suggesting that methylation has a negative effect expression of these genes.

Consistent results were observed in the study by Nakagawa T. et al., where ZNF683 was significantly hypomethylated in the cancer cohort (*p* = 0.02) (Figure 9A). Their data also showed hypermethylation of ITK promoters in normal tissue (*p* = 0.09), and no significant difference in ATF2 methylation between the two groups (Table 5). We also visualised methylation status of CpG promotors affecting the expression of the three genes of interest (Figure 9B). We observed that patients with highly methylated genes predominantly had lower NKT ES, which also might indicate an epigenetic regulatory effect (Figure 10).

### 3.4. Macrophage Activation and Clinical Data

**Figure 11:**
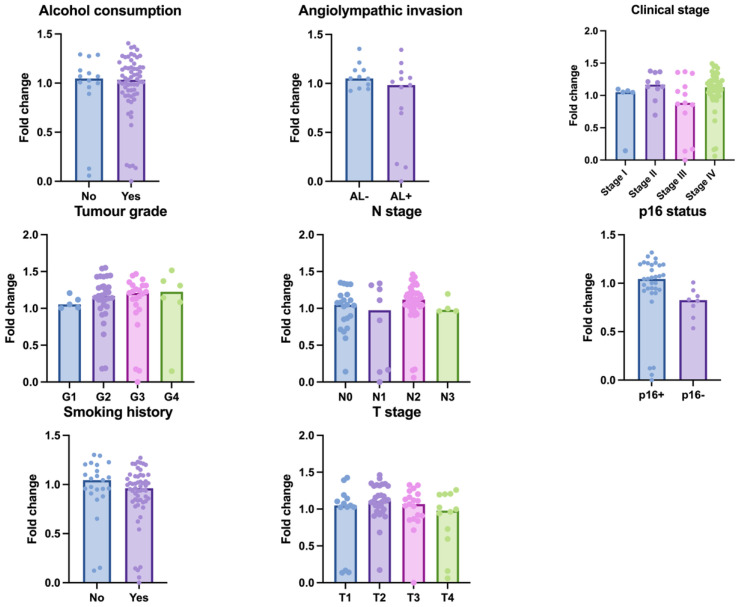
The figure shows macrophage activation enrichment scores (expressed as fold change) across several clinical and pathological factors in the patient cohort.

We analysed macrophage activation ES with clinical data. These patients present commonly in higher T, N, and clinical stages. We observed a 1.28-fold-higher changes in the p16+ group (*p* = 0.0135), implying macrophage activation in HPV+ OPSCC (Figure 11).

### 3.5. Deconvolution and Interleukin Expression

**Figure 12:**
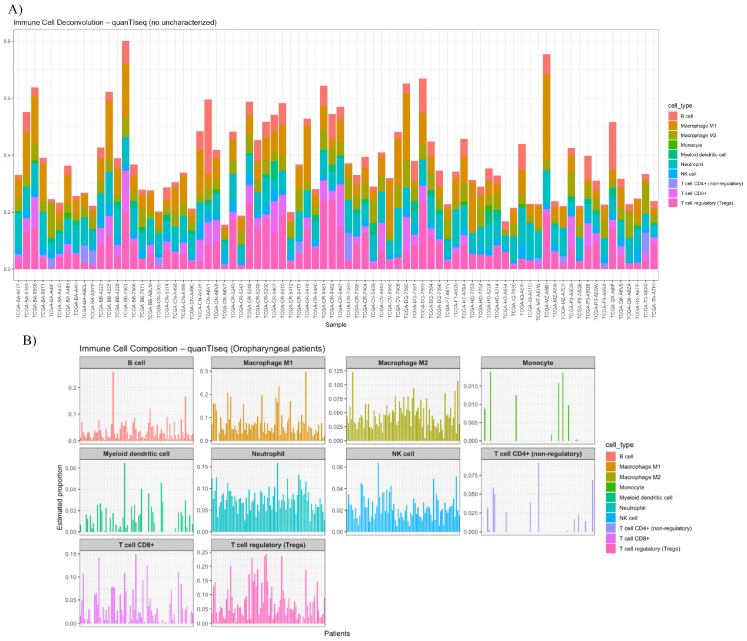
Deconvolution analysis of TCGA samples: (**A**) Bar plot showing estimated proportions of immune cell subsets across OPSCC samples based on quanTIseq deconvolution. Each stacked bar represents an individual tumour, with colours indicating different immune cell types; (**B**) individual immune cell fractions presented for every sample.

**Figure 13:**
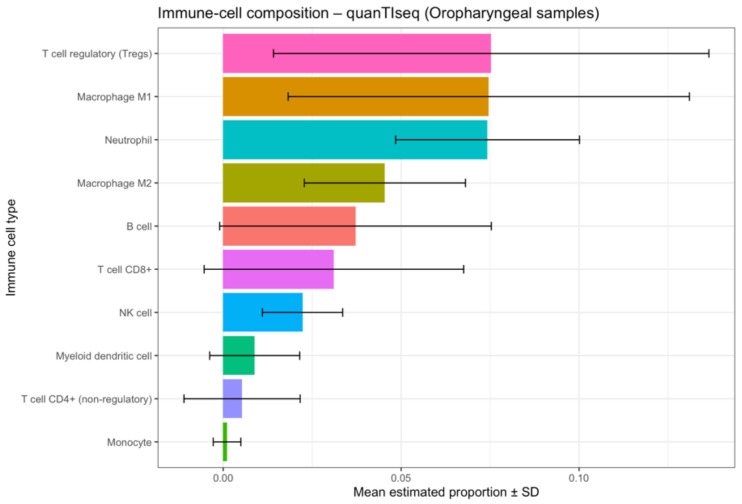
Bar plot summarising the mean estimated proportions (±SD) of immune cell subsets from quanTIseq analysis. T regulatory cells (Tregs), M1 macrophages, and neutrophils represent the predominant immune populations within OPSCC tumours.

In order to better understand the immune cell interactions, we performed quanTIseq deconvolution analysis (Figure 12), where we approximated the number of immune cells in our samples. Our study found an overall prevalence of regulatory T lymphocytes (Treg), followed by M1 macrophage and neutrophiles (Figure 13).

**Figure 14:**
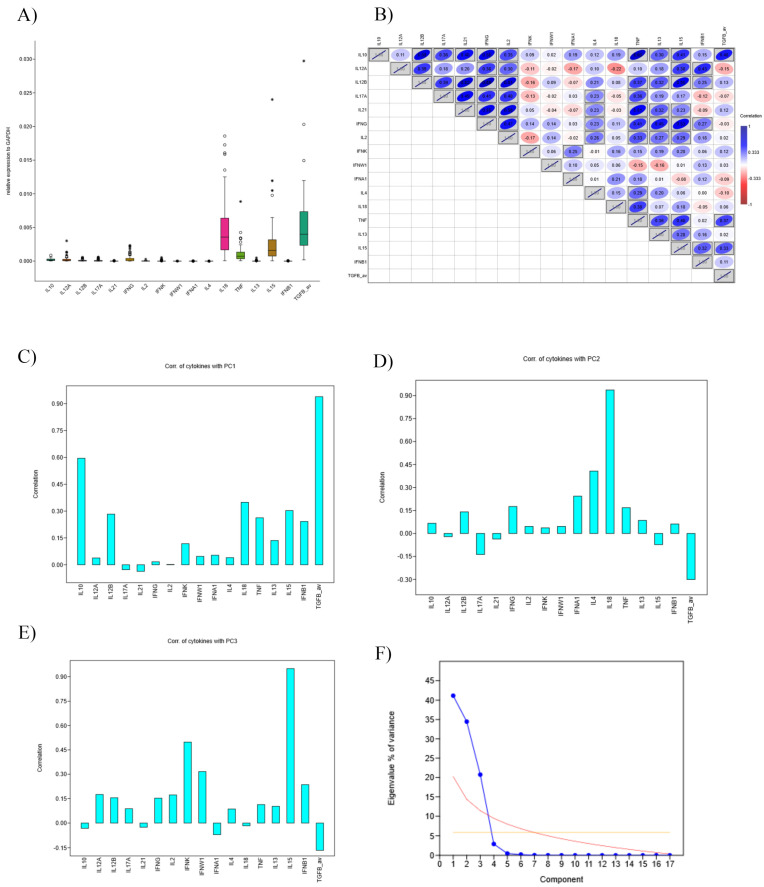
Gene expression for cytokines. (**A**) IL-15, IL-18, TNFα, TGFβ showed highest expression in OPSCC. * Stands for extreme outliers (values that fall outside 3× the interquartile range), while empty circles stand for mild outliers (values that fall outside 1.5× the interquartile range). (**B**) Correlation matrix of the cytokines, higher correlation is marked by blue, while lower is marked with red. The correlations that showed statistical significance are marked grey. (**C**) Principal component 1 (PC1) showed a strong correlation with TGFβ, highlighting the immunosuppressive nature of this group. (**D**) Principal component 2 (PC2) correlated highly with IL-18, suggesting innate activity. (**E**) Principal component 3 (PC3) showed highest correlation with IL-15. (**F**) The first three components explain more than 90% of variances, with a dominance of PC1. The steep decline after PC3 (elbow point) suggests that these three components capture the major biological variability in cytokine expression.

To further understand the potential interactions between immune cells, we also performed an analysis of cytokine expression genes in tumours; the most expressed cytokines were TGFβ, IL-18, IL-15, and TNFα (Figure 14A). Since cytokine expression correlated with each other (Figure 14B), we performed principal component analysis (Figure 14F). The first three components explained more than 90% of variance. Principal component 1 (Figure 14C) correlated very strongly with TGFβ, moderately with IL-10 and weakly with TNFα; principal component 2 (Figure 14D) correlated strongly with IL-18 and fairly with IL-4, finally the third principal component (Figure 14E) correlated strongly with IL-15 and moderately with type-I interferons (IFNK and IFNW1). In addition, components 2 and 3 inversely correlated with TGFβ. None of the principal components correlated with survival.

### 3.6. KEGG Pathway Enrichment and Gene–Pathway Network Analysis

**Figure 15:**
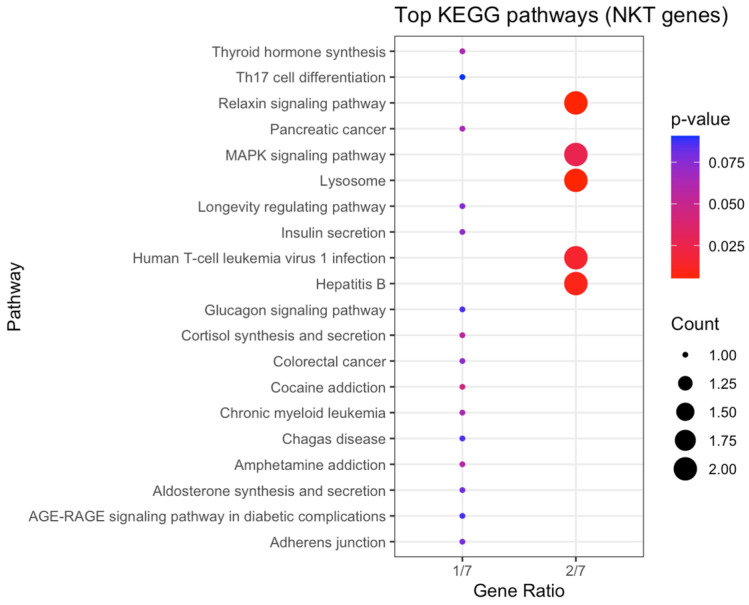
KEGG pathway enrichment analysis of genes associated with NKT cell differentiation in OPSCC. The X-axis represents the gene ratio, while bubble size indicates gene count and colour denotes adjusted *p* value.

To explore downstream molecular mechanisms associated with NKT-related genes, KEGG enrichment analysis was conducted. The top enriched pathways included MAPK signalling, Th17 cell differentiation, T-cell receptor signalling, Hepatitis B, and Relaxin signalling (Figure 15).

**Figure 16:**
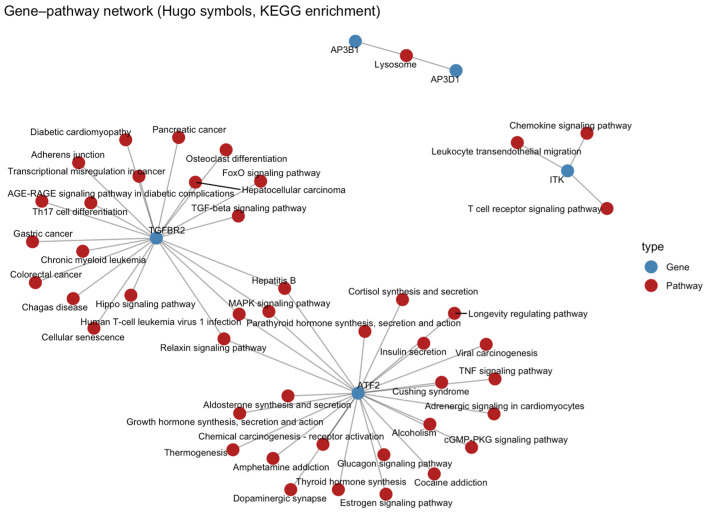
Gene–pathway interaction network displaying key NKT-associated genes (*ITK*, *ATF2*, *TGFBR2*, *AP3B1*, and *AP3D1*) and their enriched KEGG pathways. Blue nodes represent genes, and red nodes represent pathways.

A gene–pathway interaction network (Figure 16) revealed that ATF2 is connected to multiple signalling and metabolic pathways, including MAPK, TNF, and oestrogen signalling, suggesting its integrative role in stress and immune regulation. ITK is clustered with T-cell activation and cytokine-related pathways (chemokine signalling, T-cell receptor signalling), reinforcing its immunologic importance. TGFBR2 was connected to TGF-β and Th17 differentiation pathways, linking NKT activity with immune modulation and tumour suppression.

## 4. Discussion

Many studies have been carried out researching the tumour immune environment of OPSCC, primarily focusing on CD8+ and CD4+ T cell expression [20]. Other types of lymphocytes have not been as thoroughly studied. One unique population that bridges the innate and adaptive immunity are NKT cells. They are capable of direct cytotoxic activity and rapid cytokine release, playing a key role in immunosurveillance and antitumour response [21,22]. Due to their unique characteristic, NKT cells represent a specialised type of cell bridging innate and adoptive immune systems. Unlike conventional T cells, NKT cells can recognise a wide variety of antigens. Their proposed role in antitumor immunity is linked to their ability to produce IFN-γ, thereby activating NK cells and CD8+ T lymphocytes, as well as inducing IL-12 production in dendritic cells [23]. Even after adjusting for HPV status and smoking, high NKT differentiation remained an independent predictor of improved survival, highlighting its potential as a clinically meaningful biomarker beyond established prognostic factors. Given global increases in HPV-associated OPSCC and the emerging role of immune-based therapies, the findings of this study have broad relevance for biomarker development and personalised immunotherapy across diverse populations [24].

We found that NKT cell differentiation ESs were significantly associated with OS in OPSCC, suggesting that NKT activity plays a critical role in shaping patient outcomes. Supporting this finding, an in vivo study by Dhodapkar MV et al. demonstrated reversible functional defects in NKT cells among patients with progressive multiple myeloma, implicating NKT cells in the regulation of tumour growth [25]. Similarly, Molling JW et al. measured poorer overall survival outcomes among patients diagnosed with HNSCC with lower levels of circulating invariant NKT cells in 47 peripheral blood samples [26]. To our knowledge, no prior study looked at the transcriptomic expression of NKT cells in OPSCC. An immunohistochemical study conducted by Wagner S et al. described an overall better survival for patients that had a positive NK cell infiltration, compared to patients lacking NK cell infiltration [27]. Likewise, Stangl et al., in their study on HNSCC, confirmed that lower numbers of infiltrating NK cells had the highest negative predictive value on OS and disease relapse [28].

Building on these clinical and environmental findings, we described three proteins involved in the process of NKT cell differentiation significantly associated with improved OS in OPSCC: ATF2, ZNF683, and ITK.

ATF2 (Activation transcription factor 2) is a member of the basic region-leucine zipper transcription factor family and regulates numerous genes essential for cellular function. ATF2 has also been described to regulate histone H2B and H4 acetylation, regulating CRE-dependent transcription [29]. The role of ATF2 in cancer progression and outcome is controversial. For instance, Duffey et al. described that decreased expression of ATF2 in patients with HNSCC lead to an in vivo chemo resistance; interestingly, it did not affect tumour growth [30]. This is probably due to ATF2 having a role in DNA repairment. Supporting a tumour-suppressive role, Maekawa et al. demonstrated that a mouse exhibiting a heterozygote loss of *Atf2* showed an increased susceptibility for mammary tumours [31]. Contrary to these findings, other studies showed an oncogene potential in solid tumours like pancreatic carcinoma and bladder cancer [32,33]. We observed that ATF2 CpG sites were globally hypomethylated in OPSCC. However, relative increases in methylation were associated with higher ATF2 expression. Since increased ATF2 expression was associated with improved overall survival, these findings suggest that methylation at ATF2 CpG sites may act as a permissive rather than repressive epigenetic mark in OPSCC.

ZNF683 (or Hobit), is a transcription factor involved in the terminal differentiation of NKT cells [34]. Its function, however, is not only restricted to NKT cells. ZNF683 is also a key regulator of tissue-resident memory T cells (Trm), a subset of peripheral CD8+ T lymphocytes that remain localised within tissues rather than recirculating through the bloodstream. Trm cells have been shown to exert potent antitumoral activity [35,36]. Consistent results were observed in the study by Nakagawa T. et al., where ZNF683 was significantly hypomethylated in the cancer cohort. These findings support our results, which suggest that ZNF683 contributes to antitumoral immune responses.

Another protein we found to have a favourable OS is ITK (IL-2-inducible tyrosine kinase), a member of the Tec family of non-receptor tyrosine kinase necessary for T-lymphocyte development, and particularly NKT cells [37]. Various studies have confirmed the role of ITK in oncogenesis, primarily in Hodgkin and non-Hodgkin lymphoma. In 2019, a case report of two siblings with biallelic ITK mutation and HPV infection resulted in epidermodysplasia verruciformis, a precancerous lesion that can lead to skin squamous cell carcinoma and Hodgkin lymphoma. Both patients exhibited a reduced number of NKT cells in their peripheral blood [38]. Our methylation data analysis of data from Soares-Lima et al. found significant hypermethylation of ITK in cancerous tissue. Similarly, we noticed a trend of hypermethylation of ITK promotor regions in OPSCC in TCGA data. However, data from Nakagawa T. et al. showed a trend towards hypermethylation in normal tissue, although not statistically significant. In contrast to our findings, Carson et al. described hypomethylation of CpG islands within ITK in melanoma, concluding that higher expression of ITK may promote carcinogenesis [39]. Similarly to the data from Nakagawa et al., Zamora-Fuentes et al. found ITK to be hypomethylated in all stages of clear cell renal cancer, classifying ITK as methylation-related oncogene; however, contrary to Carson et al., Zamora-Fuentes’ data shows that the cohort with higher expressions of ITK has a more favourable OS [40]. Furthermore, while our methylation analysis supports epigenetic regulation of ITK, ZNF683, and ATF2, the functional consequences of these modifications remain to be experimentally validated. Importantly, such DNA methylation changes are potentially reversible, which opens avenues for targeted epigenetic modulation. Recent CRISPR-based editing systems, such as catalytically inactive dCas9 fused with TET or DNMT enzymes, allow locus-specific demethylation or methylation of CpG regions [41]. Applying these tools in OPSCC models could help reverse specific epigenetic marks and directly assess their mechanistic and therapeutic impact.

Patients with higher macrophage activation ES had a higher OS and correlated with p16 positivity. In their immunohistochemical study, Tosi et al. noticed a different effect of tumour-associated macrophage dependent on HPV status, where HPV+ cancers had a favourable effect on survival, unlike the HPV cohort [42]. Snietura et al. also found that CD68+ and CD163+ cells were more expressed in tumour, with the CD163+ phenotype exhibiting negative characteristics like lower OS and loco-regional control, especially in HPV-OPSCC [43]. Our deconvolution analysis also highlights the pivotal role macrophages have on the TME. In addition to this, cytokines involved with innate immunity and Treg response seem to be the most expressed ones. Given the heterogeneity of head and neck squamous cell carcinomas, where some subtypes display markedly more aggressive behaviour while others have more favourable survival [44], further epigenetic research should be extended to other oropharyngeal and head and neck cancer subtypes to better understand how epigenetic regulation contributes to these clinical differences. Given the predominance of innate immune cells like NK/NKT cells and macrophages, we believe that further research should be focused on their interaction in the TME, and how this communication could be used as a novel therapeutic strategy or for employing therapies that are already used in other types of cancer such as type-I interferons.

The main limitation of our study is the relatively small sample size and its retrospective design. The retrospective nature of the dataset introduces potential bias in patient selection and data completeness. Despite standardised TCGA curation, residual confounding by unmeasured clinical variables cannot be excluded. While ssGSEA enables cell-type-specific pathway quantification at the individual sample level, it cannot distinguish tumour versus stromal cell origin or account for cellular heterogeneity within bulk transcriptomic data. Functional validation through in vitro and in vivo assays will be necessary to confirm the biological role of ITK, ZNF683, and ATF2 in NKT cell differentiation and OPSCC progression. Future functional experiments (e.g., CRISPR-Cas9 knockdown or overexpression assays) are warranted to clarify how these genes interact with immune cell subsets and influence tumour progression.

## 5. Conclusions

In conclusion, our results suggest that NKT-related gene expression signatures, particularly ITK, ZNF683, and ATF2, may serve as prognostic biomarkers in OPSCC and could represent novel therapeutic targets. We also concluded that epigenetic regulation is an important mechanism in OPSCC carcinogenesis. Given that epigenetic alterations are reversible, this raises the possibility that pharmacologic reprogramming of immune-related genes could augment antitumour immunity by activating innate cells and improve responses to immunotherapy in OPSCC. However, larger and functionally oriented studies describing these mechanisms are needed to validate these findings and clarify the contributions of NKT cells in OPSCC pathogenesis.

## Figures and Tables

**Table 1:** A detailed table showing precise gene sets used in this study with their respectful medians. Log-rank tests were used to calculate *p* value, and Gehan–Breslow–Wilcoxon test was used to calculate corrected *p* value.

Gene Set Name	Low Expression (OS Median)		High Expression (OS Median)		Difference in OS Median		*p* (Log-Rank)
	mo.	y.	mo.	y.	mo.	y.	
GOBP_NK_T_CELL_DIFFERENTIATION	142.3	11.86	171.1	14.26	28.8	2.40	0.015
GOBP_MACROPHAGE_ACTIVATION_INVOLVED_IN_IMMUNE_RESPONSE	56.9	4.74	Undefined				0.0637
GOBP_NEUTROPHIL_ACTIVATION_INVOLVED_IN_IMMUNE_RESPONSE	117.5	9.79	144.7	12.06	27.2	2.27	0.1078
GOBP_NEGATIVE_REGULATION_OF_ALPHA_BETA_T_CELL_DIFFERENTIATION	117.5	9.79	144.7	12.06	27.2	2.27	0.1199
GOBP_NEGATIVE_REGULATION_OF_CD4_POSITIVE_ALPHA_BETA_T_CELL_DIFFERENTIATION	143.6	11.97	144.7	12.06	1.1	0.09	0.1347
GOBP_REGULATION_OF_NEUTROPHIL_MEDIATED_CYTOTOXICITY	171.1	14.26	117.5	9.79	−53.6	−4.47	0.1363
GOBP_NEGATIVE_REGULATION_OF_CD4_POSITIVE_ALPHA_BETA_T_CELL_ACTIVATION	143.6	11.97	144.7	12.06	1.1	0.09	0.1408
GOBP_NATURAL_KILLER_CELL_ACTIVATION_INVOLVED_IN_IMMUNE_RESPONSE	143.6	11.97	144.7	12.06	1.1	0.09	0.2272
GOBP_REGULATION_OF_NATURAL_KILLER_CELL_MEDIATED_IMMUNE_RESPONSE_TO_TUMOR_CELL	117.5	9.79	144.7	12.06	27.2	2.27	0.2787
GOBP_NEGATIVE_REGULATION_OF_REGULATORY_T_CELL_DIFFERENTIATION	143.6	11.97	144.7	12.06	1.1	0.09	0.2955
GOBP_NEGATIVE_REGULATION_OF_ALPHA_BETA_T_CELL_ACTIVATION	143.6	11.97	144.7	12.06	1.1	0.09	0.3196
GOBP_NK_T_CELL_PROLIFERATION	117.5	9.79	144.7	12.06	27.2	2.27	0.3261
GOBP_REGULATORY_T_CELL_DIFFERENTIATION	143.6	11.97	144.7	12.06	1.1	0.09	0.3296
GOBP_NEGATIVE_REGULATION_OF_CD4_POSITIVE_ALPHA_BETA_T_CELL_PROLIFERATION	143.6	11.97	144.7	12.06	1.1	0.09	0.3788
GOBP_NEGATIVE_REGULATION_OF_ALPHA_BETA_T_CELL_PROLIFERATION	143.6	11.97	144.7	12.06	1.1	0.09	0.397
GOBP_NEGATIVE_REGULATION_OF_CD8_POSITIVE_ALPHA_BETA_T_CELL_ACTIVATION	142.3	11.86	144.7	12.06	2.4	0.20	0.541
GOBP_NK_T_CELL_ACTIVATION	142.3	11.86	144.7	12.06	2.4	0.20	0.7386

**Table 2:** Bias-reduced discrete-time survival model using a complementary log-log (cloglog) link.

Discrete-Time Survival (Cloglog)		
Bias-reduced hazard ratios (Firth-like)		
Variable	HR (95% CI)	*p*
Intercept	0.02 (0.00–0.47)	0.014
NKT High vs. Low	0.13 (0.03–0.49)	0.003
HPV Positive vs. Negative	1.34 (0.43–4.17)	0.612
Smoking Yes vs. No	0.69 (0.29–1.66)	0.41

**Table 3:** Survival associations of candidate genes within the NKT cell differentiation signature in OPSCC.

Gene Name	Low Median	High Median	*p* (Log-Rank)
*ITK*	93.13	171.1	0.0001
*ZNF683*	143.6	144.7	0.0188
*ATF2*	142.3	171.1	0.0336
*TGFBR2*	144.7	143.6	0.0959
*PRDM1*	142.3	144.7	0.1599
*TOX*	142.3	171.1	0.3135
*AP3B1*	144.7	143.6	0.535
*AP3D1*	144.7	143.6	0.7805
*ZBTB7B*	171.1	142.3	0.9916

**Table 4:** Differential methylation analysis of ATF2, ITK, and ZNF683 in OPSCC.

Gene	Mean M Normal	Mean M Tumour	Delta M	Fold Change	t Stat	*p* Value
*ITK*	−0.83	−0.2859256	0.55	1.46	3.25	0.003
*ZNF683*	0.96	0.84	−0.11	0.93	−1.15	0.26
*ATF2*	−2.47	−2.46	0.01	1.004	0.09	0.93

**Table 5:** Differential methylation analysis of ATF2, ITK, and ZNF683 in OPSCC tumour tissue versus normal mucosa [19].

Gene	Mean M Mucosa	Mean M Cancer	Delta M	Fold Change	t Stat	*p* Value
*ZNF683*	0.66	0.17	−0.49	0.71	−2.80	0.02
*ITK*	0.33	−0.27	−0.59	0.66	−1.97	0.09
*ATF2*	−3.79	−3.60	0.19	1.14	0.41	0.71

## Data Availability

Datasets generated and/or analysed during the current study are available upon reasonable request from the corresponding author.

## Abbreviations

The following abbreviations are used in this manuscript:

**Abbreviation**	**Full Term**
AJCC	American Joint Committee on Cancer
ANOVA	Analysis of Variance
ATF2	Activating Transcription Factor 2
CI	Confidence Interval
cloglog	Complementary Log-Log
CRE	cAMP Response Element
GEO	Gene Expression Omnibus
GOBP	Gene Ontology Biological Process
H2B	Histone 2B
HNC	Head and Neck Cancer
HNSCC	Head and Neck Squamous Cell Carcinoma
HPV	Human Papillomavirus
HR	Hazard Ratio
IFN	Interferon
IL	Interleukin
ITK	IL2-Inducible T-cell Kinase
KEGG	Kyoto Encyclopedia of Genes and Genomes
KM	Kaplan–Meier
LAT	Linker for Activation of T cells
MAPK	Mitogen-Activated Protein Kinase
MHC	Major Histocompatibility Complex
MSigDB	Molecular Signatures Database
NK	Natural Killer
NKT	Natural Killer T (cell)
OPSCC	Oropharyngeal Squamous Cell Carcinoma
OS	Overall Survival
PCA	Principal Component Analysis
PD-1	Programmed Cell Death Protein 1
PD-L1	Programmed Death-Ligand 1
RNA-Seq	RNA Sequencing
RSEM	RNA-Seq by Expectation-Maximisation
ssGSEA	Single-Sample Gene Set Enrichment Analysis
TCGA	The Cancer Genome Atlas
TCR	T-cell Receptor
TGFβ	Transforming Growth Factor Beta
TGFBR2	Transforming Growth Factor Beta Receptor 2
Th17	T Helper 17 (cell)
TIM	Tumour Immune Microenvironment
TMB	Tumour Mutational Burden
TNFα	Tumour Necrosis Factor Alpha
TME	Tumour Microenvironment
Trm	Tissue-Resident Memory (T cell)
Treg	Regulatory T cell
WAS	Wiskott–Aldrich Syndrome protein
ZNF683	Zinc Finger Protein 683 (Hobit)
